# Serum extracellular vesicle depletion processes affect release and infectivity of HIV-1 in culture

**DOI:** 10.1038/s41598-017-02908-5

**Published:** 2017-05-31

**Authors:** Zhaohao Liao, Dillon C. Muth, Erez Eitan, Meghan Travers, Lisa N. Learman, Elin Lehrmann, Kenneth W. Witwer

**Affiliations:** 1 Department of Molecular and Comparative Pathobiology, The Johns Hopkins University School of Medicine, Baltimore, MD United States; 2 Department of Neurology, The Johns Hopkins University School of Medicine, Baltimore, MD United States; 3 Cellular and Molecular Medicine Program, The Johns Hopkins University School of Medicine, Baltimore, MD United States; 4 Laboratory of Neurosciences, National Institute on Aging, National Institutes of Health, 251 Bayview Boulevard, Baltimore, MD 21224 United States

## Abstract

Extracellular vesicles (EVs) are involved in intercellular communication and affect processes including immune and antiviral responses. Blood serum, a common cell culture medium component, is replete with EVs and must be depleted prior to EV-related experiments. The extent to which depletion processes deplete non-EV particles is incompletely understood, but depleted serum is associated with reduced viability and growth in cell culture. Here, we examined whether serum depleted by two methods affected HIV-1 replication. In cell lines, including HIV-1 latency models, increased HIV-1 production was observed, along with changes in cell behavior and viability. Add-back of ultracentrifuge pellets (enriched in EVs but possibly other particles) rescued baseline HIV-1 production. Primary cells were less sensitive to serum depletion processes. Virus produced under processed serum conditions was more infectious. Finally, changes in cellular metabolism, surface markers, and gene expression, but not miRNA profiles, were associated with depleted serum culture. In conclusion, depleted serum conditions have a substantial effect on HIV-1 production and infectivity. Dependence of cell cultures on “whole serum” must be examined carefully along with other experimental variables, keeping in mind that the effects of EVs may be accompanied by or confused with those of closely associated or physically similar particles.

## Introduction

Extracellular vesicles (EVs) are a diverse group of bilayer-membraned particles that include so-called “exosomes” (canonically defined as budding into multivesicular bodies (MVBs) and being released upon MVB fusion with the plasma membrane) and “microvesicles” (often described as blebbing directly from the plasma membrane)^1, 2^. The mode diameter of EVs in circulation approximates that of retroviral particles^1^, and EVs and retroviruses share many common features^3–5^. Indeed, HIV has been called a “Trojan exosome,” eluding the host immune responses in part by masquerading as an EV^3^.

The relationship between EVs and HIV-1 infection is an area of active study, with some contrasting findings. While several other viruses can replicate via viral genomes packaged into host EVs^6, 7^, HIV-1 does not appear to be capable of transmitting infection through this route^8^. However, EVs produced by HIV-1-infected cells contain fragments of viral RNA^9^ and viral proteins such as Nef^10^ and Gag^11^ (although another study did not find Nef to be associated with EVs)^12^. HIV infection may alter the number and size of EVs as well as the host microRNA and proteins contained in EVs, which in turn may have implications for immune activation and HIV-1 pathogenesis^13–16^. In the setting of HIV-1 infection, EVs containing viral or host components may contribute to or exacerbate other conditions, such as HIV-1- or opiate-mediated neuron damage^17, 18^. Whether specific EVs enhance or oppose HIV infection remains unclear and likely is context-dependent. EVs from HIV-infected cells can facilitate infection by several different mechanisms: by forming aggregates that include and deliver HIV-1 virions^19^; by activating CD4+ T lymphocytes, rendering them permissive for HIV-1 infection^20–22^; and by activating latent HIV-1 infection^23^. On the other hand, EVs from CD4+ T-cells can act as decoys to prevent infection of cells^16^, while EVs derived from human semen appear to inhibit HIV-1 replication and transmission^24, 25^.

We previously showed that many cell types grow more slowly in media prepared with serum that had been ultracentrifuged to remove EVs^26^. Serum EV depletion has been observed to alter cell migration^27^, and macrophages become more proinflammatory when grown without serum EVs^28^. In general, we observed a slight but significant decline in replication and viability in EV-depleted conditions^26^. The magnitude of these effects was variable, and, notably, a primary glioblastoma cell line (U87) did not appear to be affected. Adding concentrated EVs back to the EV-depleted medium rescued cell growth, suggesting that EV depletion may contribute to the reduction in cell growth. We also found that the majority of the EVs that were internalized by cells in a protein-dependent fashion were targeted to lysosomes^26^. The identity of any specific growth-promoting factors contained in the EVs, such as RNA, proteins, or lipids, remains unknown, as does the extent to which these factors are involved in nutrition, signaling, and/or information exchange.

It is important to note that the current evidence does not rule out a role for EV-associated or otherwise co-purifying factors in the findings on serum depletion. The physical processes that are meant to deplete EVs (including ultracentrifugation) surely also deplete various protein and lipid entities^29^. Although we refer to “EVD” or “EV-depleted” serum in this manuscript, we urge the reader to keep in mind that the “EV depletion” outcome of these processes may not be the only one.

Prompted by the previous findings on the effects of depleted serum, we sought to determine whether serum depletion processes might affect HIV-1 replication *in vitro*. We used serum depleted by two methods and examined the effects of media prepared with these sera on the growth and behavior of cells that are susceptible to or infected with HIV-1, including immortalized cell lines, HIV-1 latency models, and primary T-cells and monocyte-derived macrophages. We also assessed the influence of serum depletion on virus production and infectivity. Finally, possible molecular and cellular explanations for these results were probed, including cellular respiration and miRNA and mRNA expression. We conclude that two distinct serum depletion protocols—meant to remove EVs—have a profound effect on HIV-1 replication and infection in cultures of some cells, and that bulk EVs in serum, and/or their closely associated or co-depleted factors, tend to exert a virus-suppressive effect.

## Methods

### EV Depletion

To prepare “UC-EVD” (ultracentrifugation EV-depleted) FBS, FBS from Thermo Fisher (Gibco) was diluted 1:4^30^ with Dulbecco’s PBS or base culture medium and was centrifuged in a Beckman ultracentrifugation tube at 110,000 × g for 18 hours at 4 °C (*AH-629 rotor*, *k factor* = *242*). Supernatant was gently removed from the top down by pipette, avoiding disturbing the bottom of the tube, and used for preparation of EV-depleted media, which was then filtered through a 0.22 µm filter (Millex no. SLG5033SS). “TF-EVD” medium was prepared with Thermo Fisher Gibco™ Exosome-Depleted FBS (Thermo Fisher, USA, Catalog #A2720801), depleted by the manufacturer using a proprietary method. “EVR” refers to EV-replete medium.

### Single particle tracking analysis

Extracellular particle concentration was measured using a NanoSight NS300 (Malvern, Worcestershire, UK) equipped with a 532 nm laser or a NanoSight NS500 with a 405 nm laser. At least five 20 second videos were recorded of each sample at a camera setting of 10, and files were analyzed at a detection threshold of five using NanoSight NTA software version 3.1.

### Western Blot

Samples were lysed with RIPA buffer (Thermo Fisher, #89900). Protein concentration was determined by bicinchonic acid assay. 100 µg of protein from each sample was loaded onto a Criterion 10% Tris-HCl gel (Bio-Rad, Hercules, CA, cat #3451018) and electrophoresed. The proteins were then transferred to a PVDF membrane (Bio-Rad, cat #1620174) and blocked with 5% powdered milk (Bio-Rad, cat #1706404) in Dulbecco’s PBS (Thermo Fisher, cat #14190250) +0.1%Tween®20 (Sigma-Aldrich, St. Louis, MO, cat #P9416) for 1 h. The membrane was subsequently incubated with mouse-anti-human CD63 primary antibody (BD, cat #556019), at a 1:1000 dilution for 1 hour, and mouse-anti-human CD81 (Santa Cruz Biotechnology, cat #sc-166029), at a 1:1000 dilution for 1 hour. After washing the membrane, it was incubated with a goat-anti-mouse IgG-HRP secondary antibody (Santa Cruz Technology, cat #sc-2005) at a 1:10,000 dilution for 1 h. The membrane was then incubated with a 1:1 mixture of SuperSignal West Pico Stable Peroxide solution (Thermo Fisher, cat #34080) and Luminol Enhancer solution (Thermo Fisher, cat #34080) for 5 min. The membrane was visualized on Amersham Hyperfilm^TM^ ECL chemiluminescence film (GE Life Sciences, PA, cat #28906839).

### Cell Culture

#### Cell lines

H9 and PM1 (T-lymphocytic lines); the chronically HIV-1-infected T-lymphocytic (ACH-2) and promonocytic (U1) cell lines; and TZM-bl and HEK-293T cells were obtained from AIDS Reagent and ATCC. Cells were grown in complete RPMI medium (R10) prepared with EVR, UC-EVD, or TF-EVD FBS, 1 mM L-glutamine (Thermo Fisher, MA, USA; Cat #25030081), 1 mg/mL Pen-Strep (Thermo Fisher, MA, USA; Cat #15140148), and 10 mM HEPES (Thermo Fisher, MA, USA; Cat #15630080).

#### Primary cells

Blood was obtained from healthy human donors under a university-approved protocol (JHU IRB #CR00011400). Within 15 minutes of draw, blood was diluted approximately 2:5 in PBS/5 mM ethylenediaminetetraacetic acid (EDTA)/2% EV-depleted FBS, layered gently over room temperature Ficoll (GE Healthcare Biosciences, MA, USA; Cat #17-1440-03) in SepMate^TM^ 50 mL tubes (StemCell, Vancouver, Canada; Cat #85450), and centrifuged at 1200 × g for 10 minutes at room temperature. The plasma/PBMC fraction was centrifuged at 300 × g for 8 minutes and incubated in red blood cell lysis buffer (4.15 g NH_4_CL, 0.5 g KHCO_3_, 0.15 g EDTA in 450 mL H_2_O; pH adjusted to 7.2–7.3; volume adjusted to 500 mL and filter-sterilized) at 37 °C for 10 minutes. For monocyte-derived macrophages (MDM), PBMC were pelleted at 400 × g for 6 minutes at room temperature, resuspended in cell culture media, and plated at 10^7^ cells per well in 6-well plates. Differentiation proceeded for seven days in the presence of macrophage colony stimulating factor (M-CSF) (R&D Systems, MN, USA; cat #216-MC) as described previously^31^. CD4+ T-cells were isolated from PBMCs via EasySep^TM^ Human Naïve CD4+ T cell Isolation Kit (Vancouver, CA; Cat #19155) (2). Purity was determined by flow cytometry (BD Fortessa) using a CD4+ antibody (Becton Dickinson, NJ, USA; Cat #562658). Recombinant Interleukin-2 (Thermo Fisher, #PHC0026) was added as a baseline stimulant at a concentration of 10 U/mL. Cells were activated 24 hours after isolation with phytohemagglutinin (PHA) at a concentration of 5 µg/mL, diluted in culture medium (Sigma-Aldrich, MO, USA; Cat #693839-1G).

### HIV-1 Infection

HIV-1. Rf and BAL stocks were generated from infected H9 and PM1 T-lymphocytic cell lines, respectively, and stored at −80 °C. For experiments involving H9 and PM1 cultures, HIV. Rf or HIV. BAL were added at a concentration of 500 ng/mL (p24) and incubated at 37 °C for 4–6 hours. Cells were then rinsed several times with PBS and spun twice at 400 × g for 5 minutes each. For primary macrophage cultures, HIV. BAL was added at a concentration of 200–250 ng/mL (p24) and incubated overnight at 37 °C before rinsing twice with PBS.

### Cell Viability

Cell viability was assessed using the Muse™ Cell Analyzer and the Muse™ Count and Viability Kit according to manufacturer’s instructions (EMD Millipore, Billerica, MA, USA; Cat #MCH100102) or the WST-1 cell viability assay (Roche, IN, USA; Cat #5015944001). The plates were mixed on an Orbital Shaker on setting 2 (Bellco, NJ, USA; Cat #7744-20220) at room temperature for 30 min and absorbance was measured at 630 nm using an iMark™ Microplate Absorbance Reader (Bio-Rad, CA, US). MTT cell viability assay (Thermo Fisher MA, USA; cat #V-13154) was performed by incubating culture plates with MTT reagent for four hours at 37 °C, adding SDS lysis buffer, and incubating for four hours to overnight at 37 °C. Absorbance was measured at 570 nm with a plate reader as above.

### HIV-1 p24 Assay

200 µl of cleared cell culture supernatant from all evaluated conditions was stored for p24 assays (Perkin Elmer, Holland, via Thermo Fisher Cat #50-905-0509) at appropriate dilutions and following the manufacturer’s protocol. p24 levels were determined based on the manufacturer’s p24 standard. All results represent at least three separate experiments.

### Luciferase Assay

Luciferase expression in TZM-bl cells, which contain a luciferase gene under the control of a retroviral LTR, was monitored following overnight exposure of cells to H9/HIV. Rf- or PM1/HIV. BAL-containing-media from infected cells. The luciferase assay was done according to the manufacturer’s protocol using the Luciferase Assay System (Cat #E1500; Promega, WI, USA) and was read on a Fluoroskan Ascent FL luminometer (Thermo Fisher, MA, USA).

### Flow Cytometry

Cells were removed from plates by gentle pipetting, washed with 1 × PBS, and stained for 20 minutes at room temperature in the dark. All antibodies were from BD (CD4, ApC-Cy7: Cat #341095; CD18, FITC: Cat #555923; CD106, PE: Cat #555647; CD54, PE-Cy7: Cat #559771; CD195, APC: 556903). Samples were washed with 2 ml of 1x PBS once, spun at 400 × g for 5 minutes, and resuspended in 300 µl of PBS to remove excess antibodies. Using a BD LSRFortessa, PBMCs were gated on lymphocytes by forward and side scatter profiles. Data were analyzed using FlowJo software v10.1 (FlowJo, OR, USA).

### Ultracentrifuge Pellet Add-Back

EV-enriched pellets from ultracentrifuged FBS were re-suspended and added at 1/200^th^ and 1/50^th^ of the re-suspension volume to separate wells containing EVR, UC-EVD or TF-EVD serum-containing media. These pellets were co-added with virus stock at the time of infection.

### RNA Isolation And Gene Expression Analysis

HEK293T cells (5 × 10^5^) were grown on a 10 cm dish for 24 hours. Medium was replaced with fresh medium prepared with EVR or UC-EVD serum. Following 48 hours of growth, RNA was extracted using Trizol (Trizol Reagent, Invitrogen) according to manufacturer’s instructions. RNA integrity was assessed by Agilent Bioanalyzer RNA 6000 Chip (Agilent, Santa Clara, CA), and 500 ng total RNA labeled according to the manufacturer’s instructions (Illumina TotalPrep RNA kit). Biotinylated aRNA (750 ng) was hybridized to Illumina Human HT12v4 bead arrays overnight, rinsed and incubated with streptavidin-conjugated Cy3. Arrays were scanned at a resolution of 0.54 microns (Illumina iScan), and intensities were extracted from the scanned images using Illumina GenomeStudio software V2011.1. Data were normalized by Z-Score transformation and analyzed with DIANE 6.0, a spreadsheet-based microarray analysis program. Z-normalized data were analyzed with principal component analysis (PCA). Z-Scores for paired treatment groups were compared using the Z-Ratio statistic to determine gene expression changes within each comparison. Expression changes for individual genes were considered significant if they met four criteria: Z-Ratio above 1.5 or below -1.5; false detection rate (FDR) <0.30; a P-value statistic for Z-Score replicability below 0.05; and mean background-corrected signal intensity greater than zero. Gene set analysis was performed using the open-source DAVID Functional Annotation.

#### miRNA qPCR Array

Total RNA was harvested from ACH-2, U1, and MDM using the mirVana total RNA isolation protocol as previously described^32^. A custom TaqMan low density array was ordered from Thermo Fisher, containing qPCR assays for 47 common miRNAs and the snRNA U6. Reverse transcription (100 ng starting material for each condition), pre-amplification, and TLDA card processing were done using Thermo Fisher/Life Technologies reagents per manufacturer’s protocol and as previously described^33^. Data were extracted and processed as previously described^33^, except that normalization was performed to the geometric mean of ten miRNAs detected in all samples (miRs-24, 17, 30b, 106a, 142-3p, 92a, 146a, 342-3p, 21, and 16). Hierarchical clustering (Pearson, average linkage) and visualization were done with MultiExperiment Viewer (MeV)^34^.

### Respiration Assay

Macrophages were plated in a Seahorse (Seahorse Bioscience/Agilent Technologies) 96-well plate and were differentiated for seven days (see above) in EVR and EVD media. Cells were washed three times with media that did not contain sodium bicarbonate. Following one hour of incubation in a 37 °C incubator, mitochondrial activity was assessed using the Seahorse XF96 Analyzer (Seahorse Bioscience/Agilent Technologies)^35^ according to the manufacturer’s instructions. Briefly, oxygen consumption rate (OCR) was measured following sequential addition of 2 µM oligomycin, 1 µM FCCP, and 5 µM rotenone/antimycin A.

### Data Availability

Gene expression data and miRNA microarray data have been deposited with the Gene Expression Omnibus (GEO) under accessions GSE89067and GSE88838, respectively, part of the SuperSeries GSE89068 and BioProject PRJNA350212. Any additional data are available upon request.

### Statistics

For experiments with multiple groups, results were analyzed by ANOVA. For two independent variables, two-way ANOVA was performed. Appropriate multiple test corrections (Tukey’s, Sidak) were performed as indicated. For two groups compared at different time points, two-sample t-tests were used with a Holm-Sidak correction for multiple comparisons. Corrected p < 0.05 was considered significant. For analysis of gene or miRNA array results, false discovery was controlled by the method of Benjamini-Hochberg.

### Ethical Approval and Informed Consent

Primary human cells were obtained from blood donors under a healthy blood donor protocol approved by the Johns Hopkins Institutional Review Board (IRB # CR00011400). Blood products were obtained in accordance with all relevant guidelines and regulations. All donors provided informed consent.

## Results

### EV depletion processes and effects on cell line viability and proliferation

**Figure 1:**
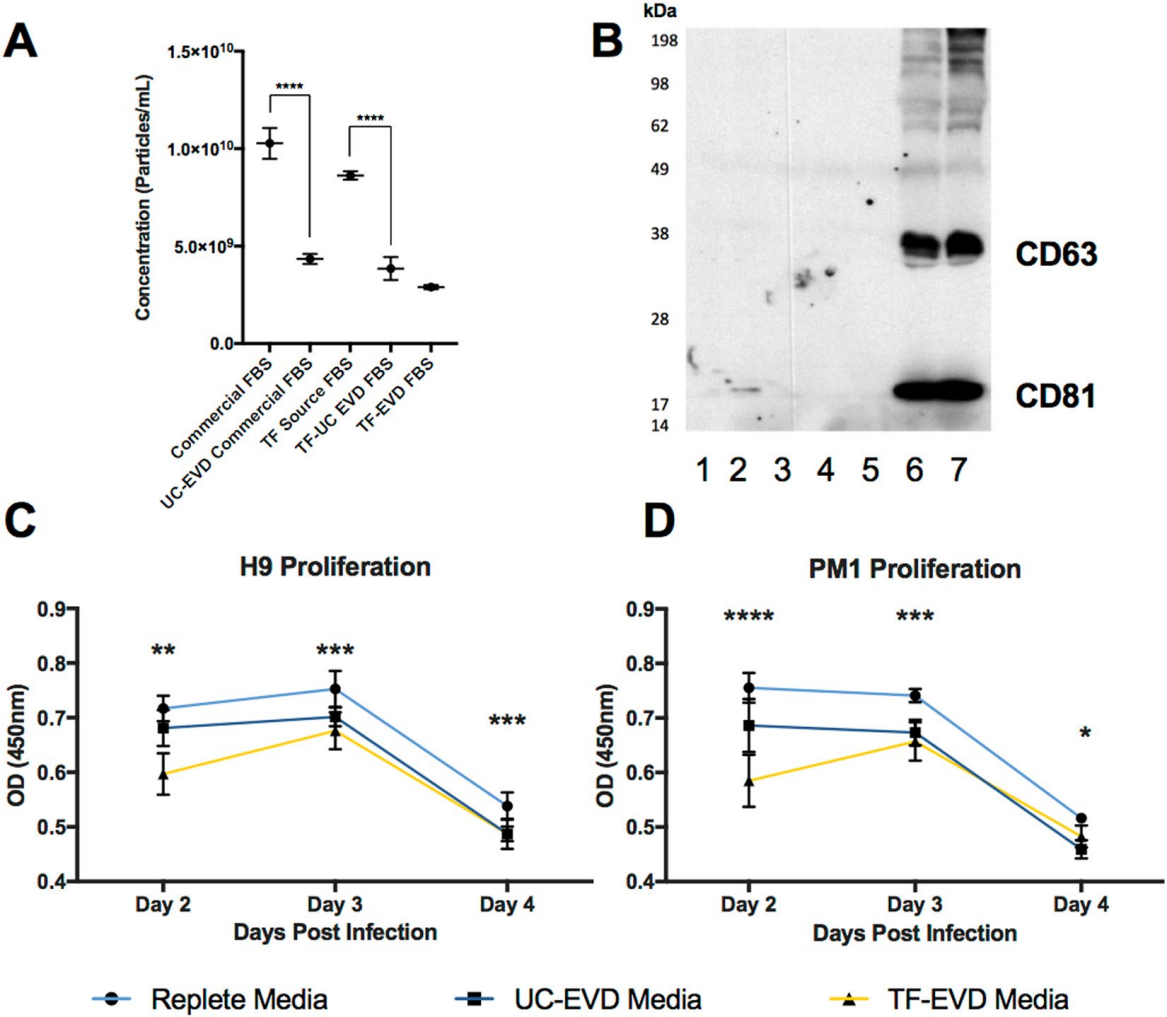
Serum particle depletion conditions affect cell proliferation. (**A**) Nanoparticle tracking comparison of particle concentrations of representative “Commercial” and “TF-Source” FBS lots with matched FBS depleted by stepped ultracentrifugation (UC) after 25% dilution; the final condition, TF-EVD, is “TF-Source” FBS depleted by Thermo Fisher using a proprietary process. The TF-EVD process achieves the greatest particle depletion. See also Figure S1 for aggregate data from additional depletions. Error bars represent standard deviation of 4 independent readings. ****p < 0.005, one-way ANOVA with Tukey’s multiple comparison test. (**B**) Anti-CD63 and –CD81 Western Blot of (1) TF-Source FBS, (2) a representative lot of another lot of FBS, and (3) TF-EVD FBS; UC supernatants of (4) TF-Source FBS and (5) other lot of FBS; and UC pellets of (6) TF Source FBS and (7) other commercial FBS. Equal protein loaded into each lane suggests overwhelming concentration of tetraspanins in the UC pellets: CD63 (MW ~30–65 kDa) and CD81 (MW ~22–25 kDa). C and D) WST-1 assay optical density for H9 (**C**) and PM1 (**D**) cultures was greatest at all time points for the replete media conditions. *p < 0.005, difference between Replete and UC-EVD only; **p < 0.005, differences between EVR and UC-EVD, UC-EVD and TF-EVD only; ***p < 0.005, differences between EVR and UC-EVD and EVR and TF-EVD media; ****p < 0.005, all types of media different from each other; Two-way ANOVA with multiple comparison correction, n = 6.

FBS was processed by dilution and overnight ultracentrifugation (“UC-EVD”) as previously described^30^ or by a proprietary commercial process (Thermo Fisher or “TF-EVD”). These sera and matched but unmanipulated FBS (“replete,” EVR) were used to make cell culture media as described in Methods. Relative abundance of particles in replete or depleted conditions was assessed by nanoparticle tracking analysis (Fig. 1A). The commercial process achieved significantly greater depletion and also showed the least variability. In contrast, ultracentrifugation-based particle depletion varied substantially by lot of serum and ultracentrifuge run (Figure S1). EV markers CD63 and CD81 were barely detected in unprocessed FBS, but were highly enriched in ultracentrifuge pellets, demonstrating EV depletion/concentration (Fig. 1B) that exceeds total particle depletion. Similar results were obtained for TSG101 and CD9 (Figure S2), but the endoplasmic reticulum marker calnexin, golgi marker GM130 and nucleus marker nucleoporin were not detected in the pelleted fraction (Figure S2). We conclude that both ultracentrifugation and the commercial process significantly but variably deplete particles including EVs from FBS, and that the TF-EVD process appears to be more effective and less variable than ultracentrifugation.

Two T-lymphocytic cell lines, H9 and PM1, were cultured in EVR, UC-EVD and TF-EVD media to assess the effect of EV-depleted serum on cell viability and growth. These lines were chosen because of their differential susceptibility to X4 and R5-tropic viruses. WST-1 assays demonstrated small but significant (p < 0.0001, 2-way ANOVA with multiple comparisons) differences in cell proliferation at Days 2, 3, 4 post initial culture conditions (Fig. 1C,D) consistent with previous reports (23). Small increases in cytotoxicity were also observed (Figure S3A). However, primary CD4+ T-cells and macrophages displayed minimal differences in metabolic activity when grown in TF-EVD media compared with replete conditions (Figure S3B).

### Increased production of HIV-1 in depleted serum conditions

**Figure 2:**
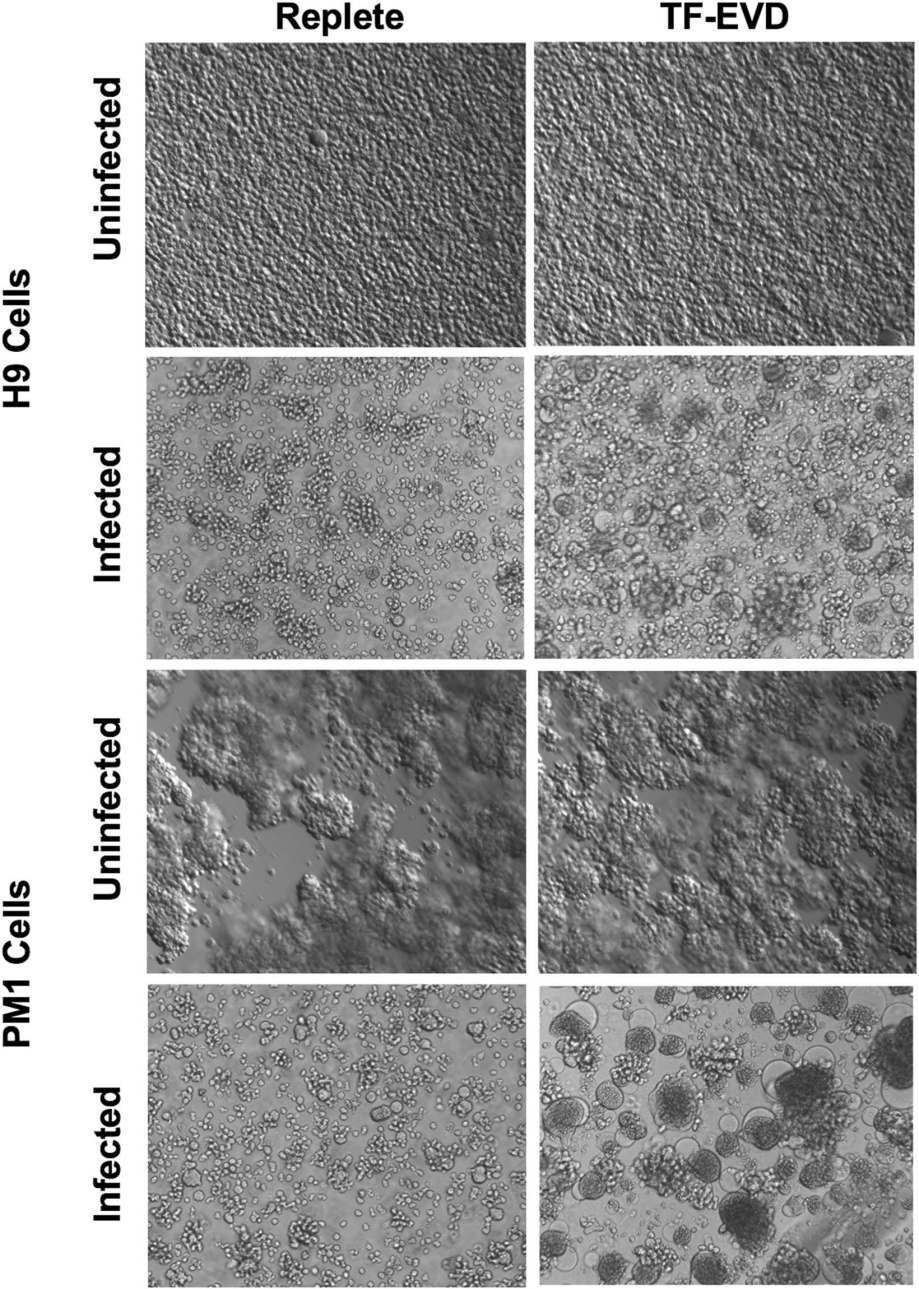
Altered cellular responses to HIV-1 in depleted serum conditions. Enhanced cell aggregation and syncytium formation were observed in cultures of HIV-1 infected H9 and PM1 cells under TF-EVD versus EVR conditions, but not in the absence of HIV-1.

**Figure 3:**
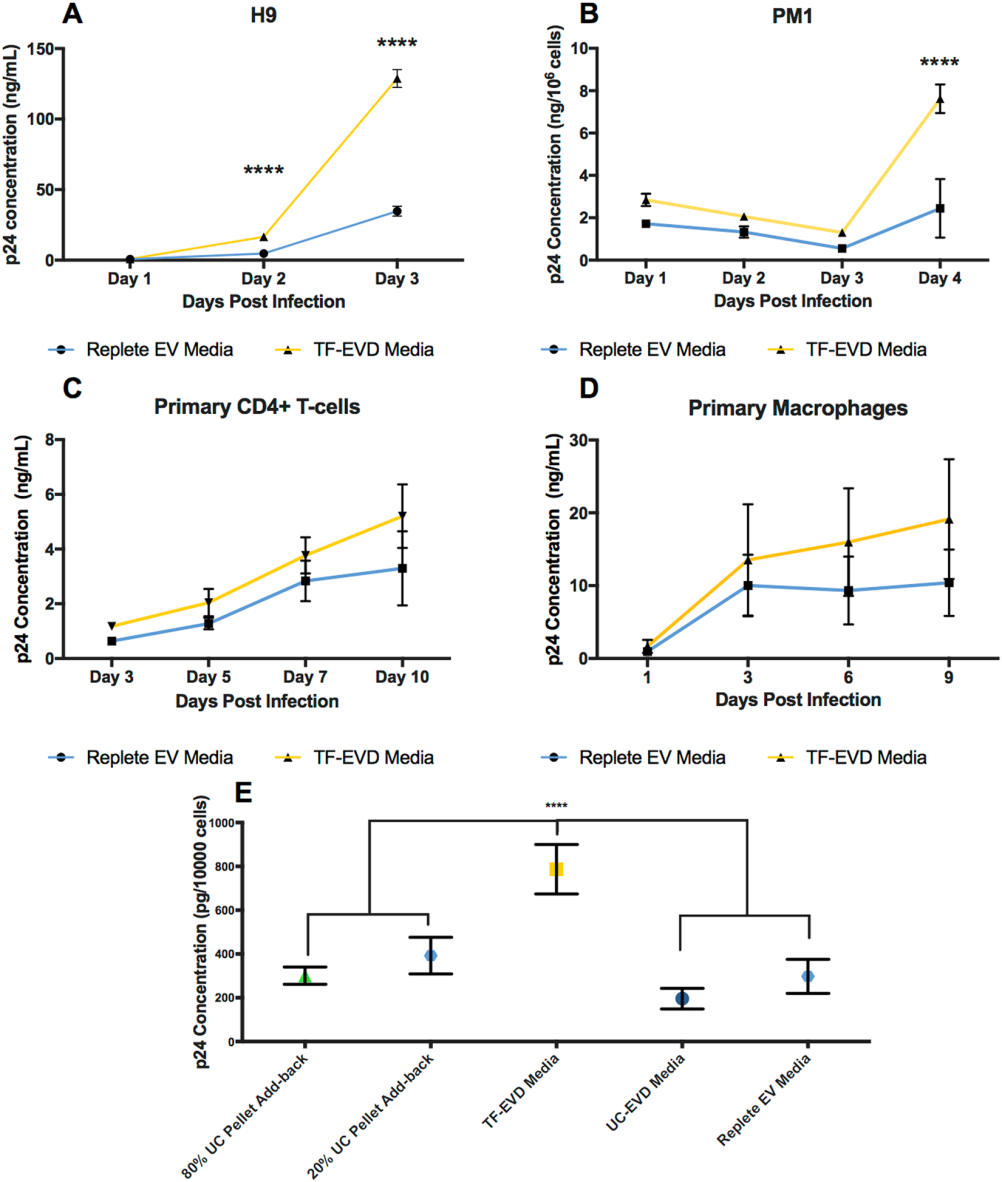
Increased p24 production in depleted serum conditions. p24 production by HIV-1. Rf-infected H9 cells (**A**) and HIV-1. BAL-infected PM1 cells (**B**) is increased significantly in TF-EVD conditions by four dpi. ****p < 0.0001, 2 sample t-test with Holm-Sidak correction (n = 3) (**B**) By Day 4 post infection, HIV. BAL production in PM1 cells grown in TF-EVD conditions is significantly higher when compared with EVR medium. ****p < 0.0001; 2 sample t-test, Holm-Sidak multiple comparison correction. Primary CD4+ T-cells infected with HIV-1. Rf (**C**) and monocyte-derived macrophages (MDM) infected with HIV-1. BAL (**D**) produced modestly higher levels of p24 when grown in TF-EVD versus replete medium (n = 3 experiments with 3 technical replicates each). (**E**) Resuspended pellets of ultracentrifuged FBS, added to TF-EVD medium at 80% and 20% of their original concentrations, depressed p24 production towards baseline in H9 culture (n = 3) at 3 dpi. ****p < 0.001, one-way ANOVA, Tukey’s multiple comparison test.

T-lymphocytic H9 and PM1 cell lines cultured for seven days under the three conditions were infected with HIV-1. Rf and HIV-1. BAL respectively. Morphologic and behavior differences were observed in the TF-EVD vs replete conditions. Specifically, infected cells (but not uninfected cells) in TF-EVD medium tended to cluster and form syncytia compared with cells in EVR medium (Fig. 2). Cells in UC-EVD medium were of intermediate phenotype that varied considerably by production lot of UC-EVD FBS, consistent with the variably efficient particle depletion that we observed using this method (Figure S1). Unexpectedly, HIV-1 release, as measured by p24 ELISA, was significantly increased in the TF-EVD condition in both H9 and PM1 cells (Fig. 3A,B). Increased HIV-1 production was also observed from primary CD4+ T-cells and MDM infected with HIV-1. Rf and HIV-1. BAL strains, respectively (Fig. 3C,D). Due to substantial donor-to-donor variability, this consistent increase did not reach significance when all experiments were combined; however, significant increases were seen for at least two post-infection time points in each individual experiment (see, e.g., MDM experiments in Figure S4).

### UC pellet add-back restores HIV-1 production to baseline levels

To confirm that depleted material (enriched in EVs, but likely containing other particulate matter) contributes to the observed effects, resuspended EV-enriched UC pellets were added back to TF-EVD culture conditions. Virus production was restored to levels significantly below the TF-EVD conditions (for example, HIV-1. Rf-infected H9 cells, Fig. 3E). That virus production was not restored completely to baseline may be consistent with reports that UC-pelleted EVs may tend to aggregate and lose functionality^36–38^. Please note that, in the set of experiments depicted in Fig. 3E, there was no significant difference between baseline and the UC-EVD condition, once again emphasizing the variability of the ultracentrifugation depletion technique and results obtained with it.

### Depleted serum cell culture conditions reverse HIV-1 latency

**Figure 4:**
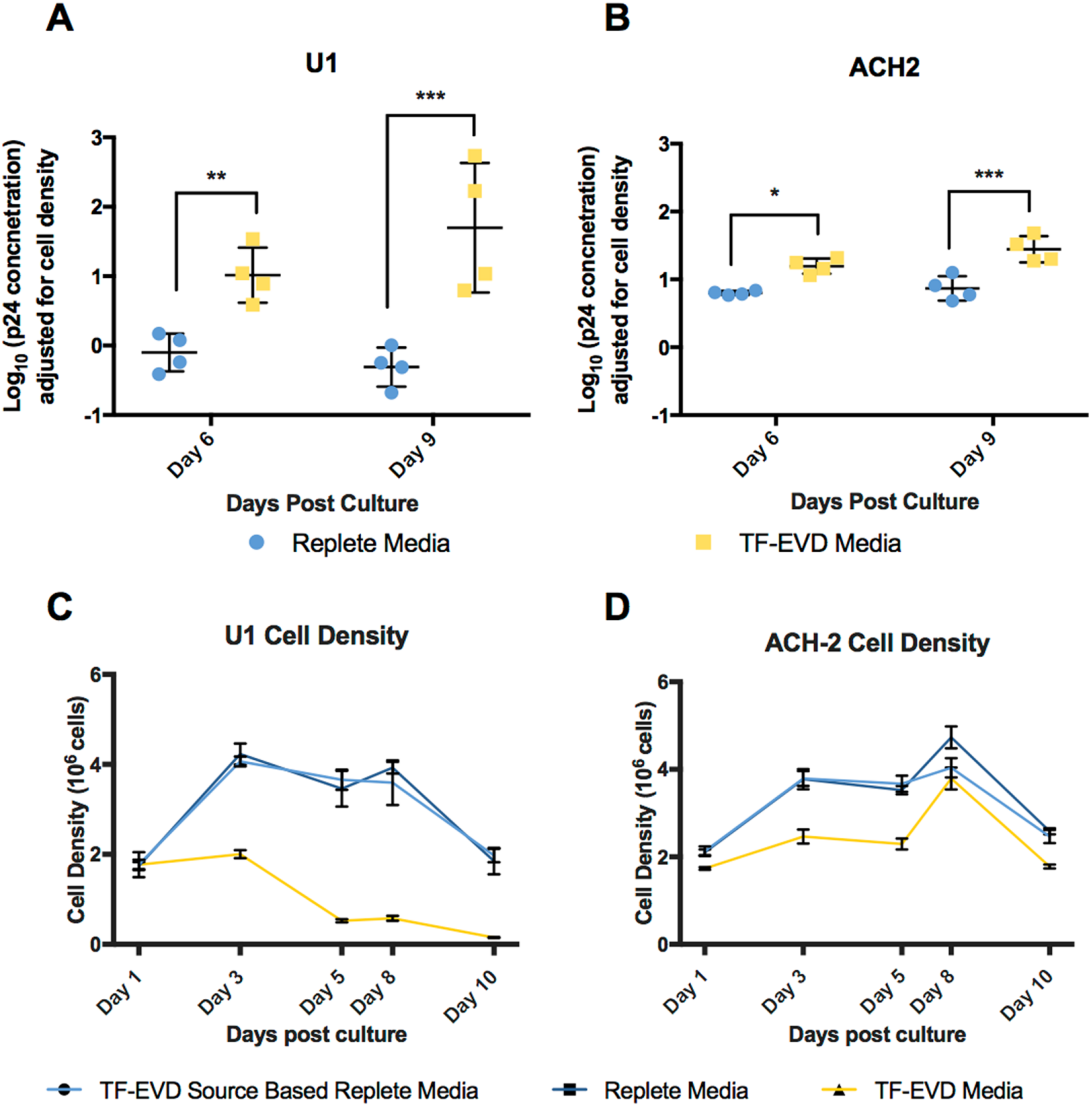
Increased p24 production in two latency models. (**A**,**B**) Greater p24 production by ACH2 and U1 cells in TF-EVD versus replete conditions (four independent experiments, n = 3 replicates each). *p < 0.05, **p < 0.005, ***p < 0.0005, two-way ANOVA with Sidak’s multiple comparison test. (**C**,**D**) Representative cell density data for U1 and ACH-2 cells from a single experiment.

Effects of serum depletion conditions on HIV-1 production could be exerted at several stages of the virus life cycle. We therefore used U1 and ACH2 cells—models of HIV-1 latency in monocyte-lineage cells and T-cells, respectively—to assess the effects of serum particle depletion on clonal populations already chronically infected with HIV-1. Cells were cultured in replete medium or TF-EVD medium conditions. Differences in p24 production were observed for both U1 and ACH2 cells (Fig. 4A and B) despite effects on cell density and viability for both cell types (Fig. 4C and D). Of interest, U1 cells were by far the most sensitive to depleted conditions of any cell type we have studied to date. Nevertheless, despite this sensitivity, increased p24 release continued even with greatly reduced cell density. This p24 detection is not simply the result of release from dead cells, as intracellular p24 in infected cells including U1 and ACH2 is lower or on par with supernatant p24 (Figures S5, and compare with results from PM1 cells) Therefore, even simultaneous release of all intracellular p24 would not explain the degree of increased p24 release observed in our serum depletion experiments, nor sustained increases over time.

### Heightened infectivity and susceptibility under depleted cell culture conditions

**Figure 5:**
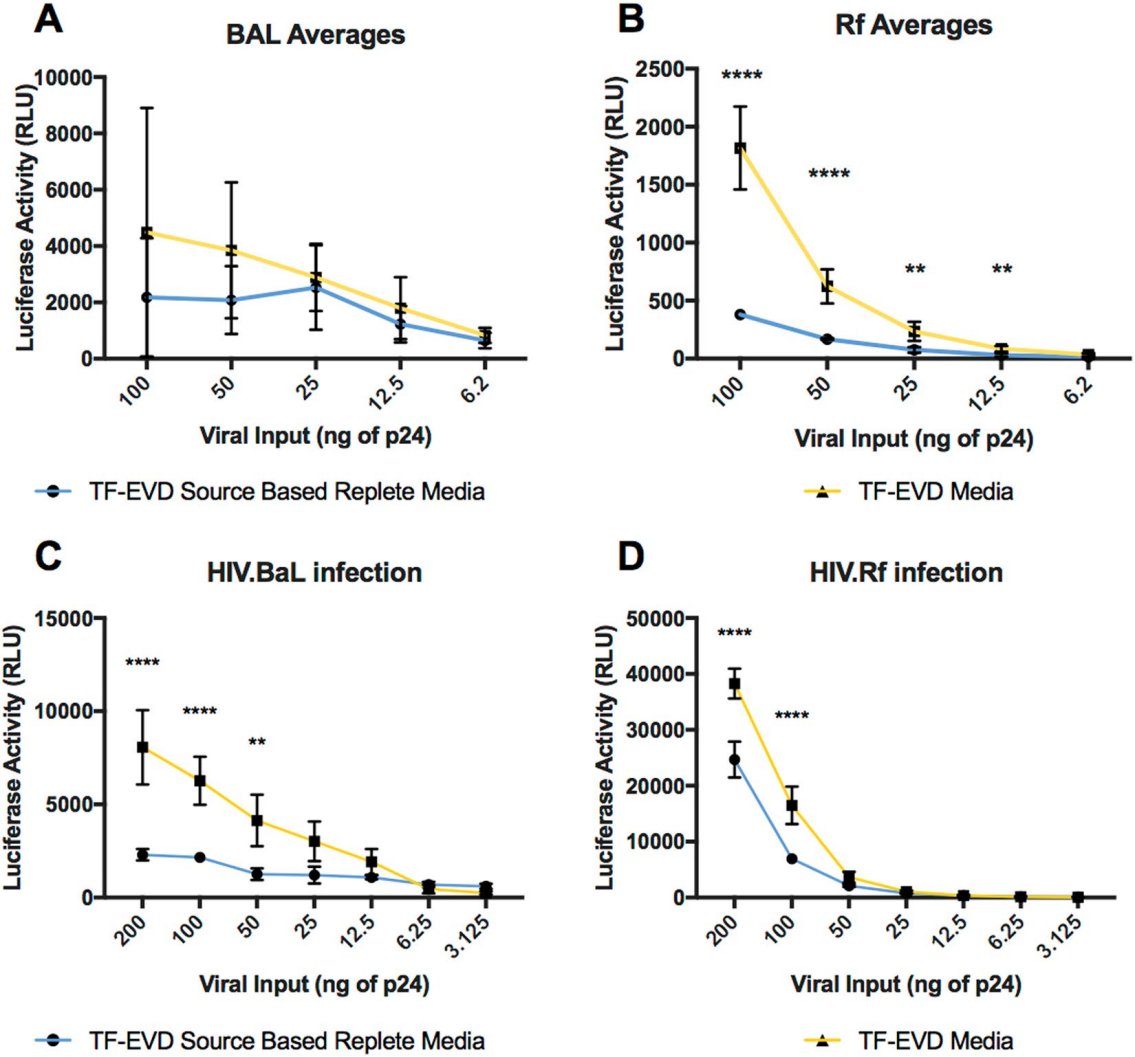
Depleted serum conditions affect infectivity of produced virus and susceptibility of uninfected cells. (**A**,**B**) HIV-1. BAL or HIV-1. Rf stocks were made from PM1 and H9 cultures, respectively, grown in EVR or TF-EVD media. Virus was collected at 7 dpi and used to inoculate TZM-bl cells at p24-normalized amounts. After overnight incubation, greater luciferase activity was observed in cells exposed to TF-EVD-produced virus (****p < 0.0001, **p < 0.01, two-way ANOVA with Sidak’s multiple comparison test, three independent experiments with at least three replicate culture wells each). Note that the lack of significance for BAL, in aggregate, belies significant differences in each individual experiment (see Figure S6). (**C**,**D**) HIV-1. BAL and RF stocks were added to TZM-bl cells grown in depleted or replete serum conditions. ****p < 0.0001, **p < 0.01, two-way ANOVA with Sidak’s multiple comparison test, n = 3.

Another stage of the viral life cycle that could be affected by serum-depleted particles including EVs is cell entry. HIV-1 produced under serum depleted conditions was collected from H9 and PM1 cells and exposed in p24-normalized amounts to TZM-bl reporter cells, which encode a luciferase gene under the control of the HIV-1 LTR. HIV-1 prepared under TF-EVD conditions drove luciferase levels at least two-fold higher compared equal amounts of virus produced under replete serum conditions for most tested concentrations of virus (Fig. 5A and B, Figure S6). Serum conditions also affected the susceptibility of recipient cells. When HIV-1. BAL and HIV-1. RF prepared in serum replete conditions was added to TZM-bl cells grown in TF-EVD media or matched, replete serum, significant differences in luciferase expression were observed (Fig. 5C and D; Figure S7).

### Assessing functional differences of cell lines raised in depleted serum conditions

To gather evidence for cellular changes that might contribute to the observed responses to serum depletion processes, we performed several tests, including flow cytometry, respiration assays, miRNA qPCR array, and gene array.

#### Flow cytometry: cell surface proteins

**Figure 6:**
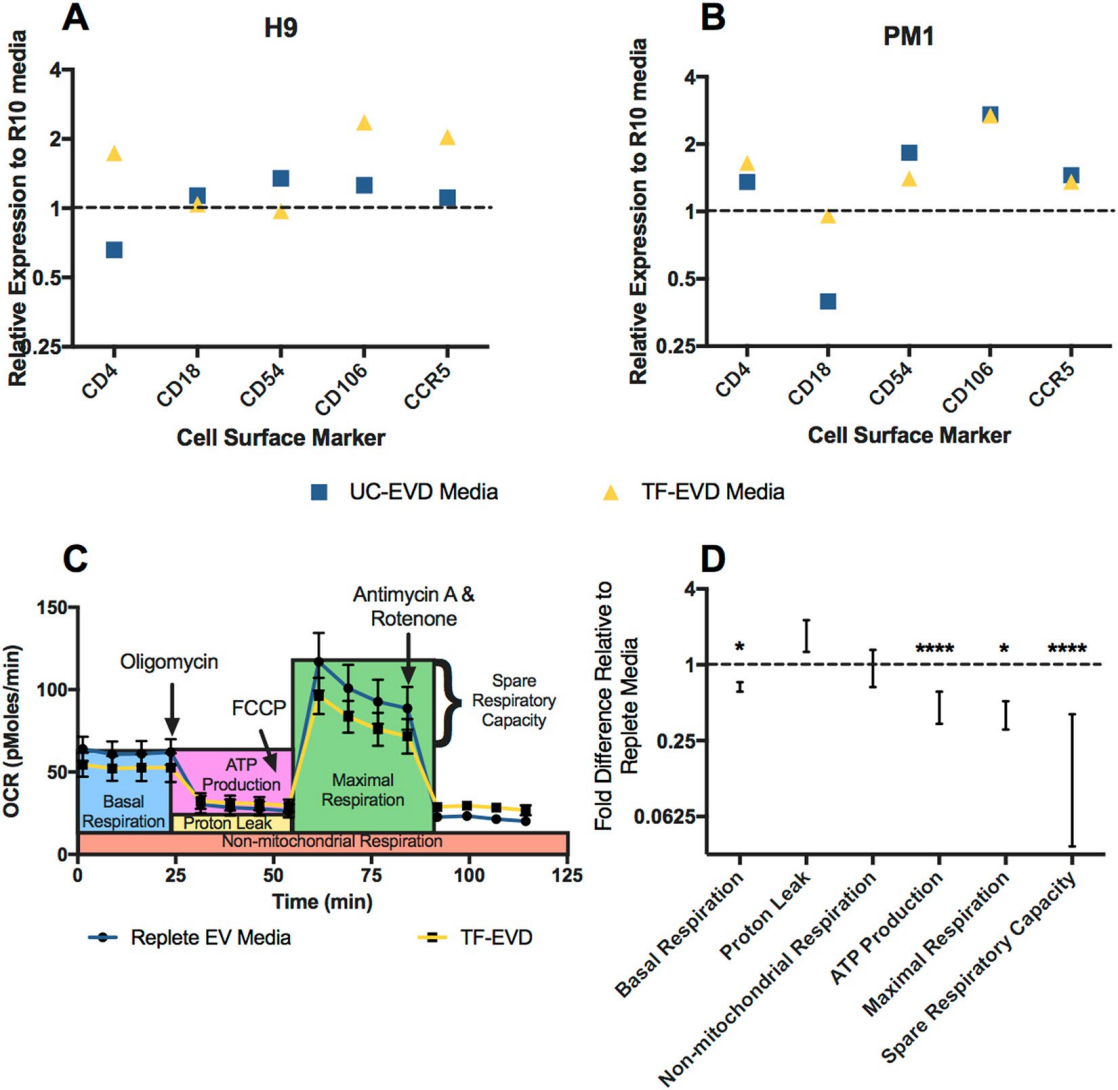
Depleted serum conditions affect cell surface markers and respiration. (**A**) H9 cells and (**B**) PM1 cells displayed increased expression of selected surface/adhesion proteins by flow cytometry when cultured in UC-EVD or TF-EVD medium for 7 days; 1 = expression on cells cultured in EVR. (**C**,**D**) Seahorse Respiration Assay of monocyte-derived macrophages grown in TF-EVD reveals significantly reduced basal and maximal respiration, as well as compromised ATP production and spare respiratory capacity. ****p < 0.001, *p < 0.05, 2-way ANOVA with multiple comparison, Sidak test. Annotations in (**C**) are adapted from the product materials (Agilent Technologies). “1” (dotted line in (**D**)) denotes average values for replete media for the same experiments.

The cell clumping observed in HIV-infected H9 and PM1 cell cultures under EVD conditions prompted us to examine expression of several adhesion molecules and HIV-1 co-receptors by flow cytometry. Indeed, among other differences, VCAM-1 (CD106) and CCR5 expression were found to be increased on H9 and PM1 cells cultured in depleted serum conditions (Fig. 6A and B).

#### Respiration assay

We next used the Seahorse respiration assay to test respiration under depleted serum conditions (Fig. 6C). Oxygen compensation analysis showed a significant, multiple-fold decrease in basal and maximal respiration, as well as compromised ATP production in mitochondria of MDM grown with depleted serum media (Fig. 6D). This reduction in mitochondrial respiration is in agreement with our previous and current observations of reduced cell growth.

#### miRNA qPCR array

**Figure 7:**
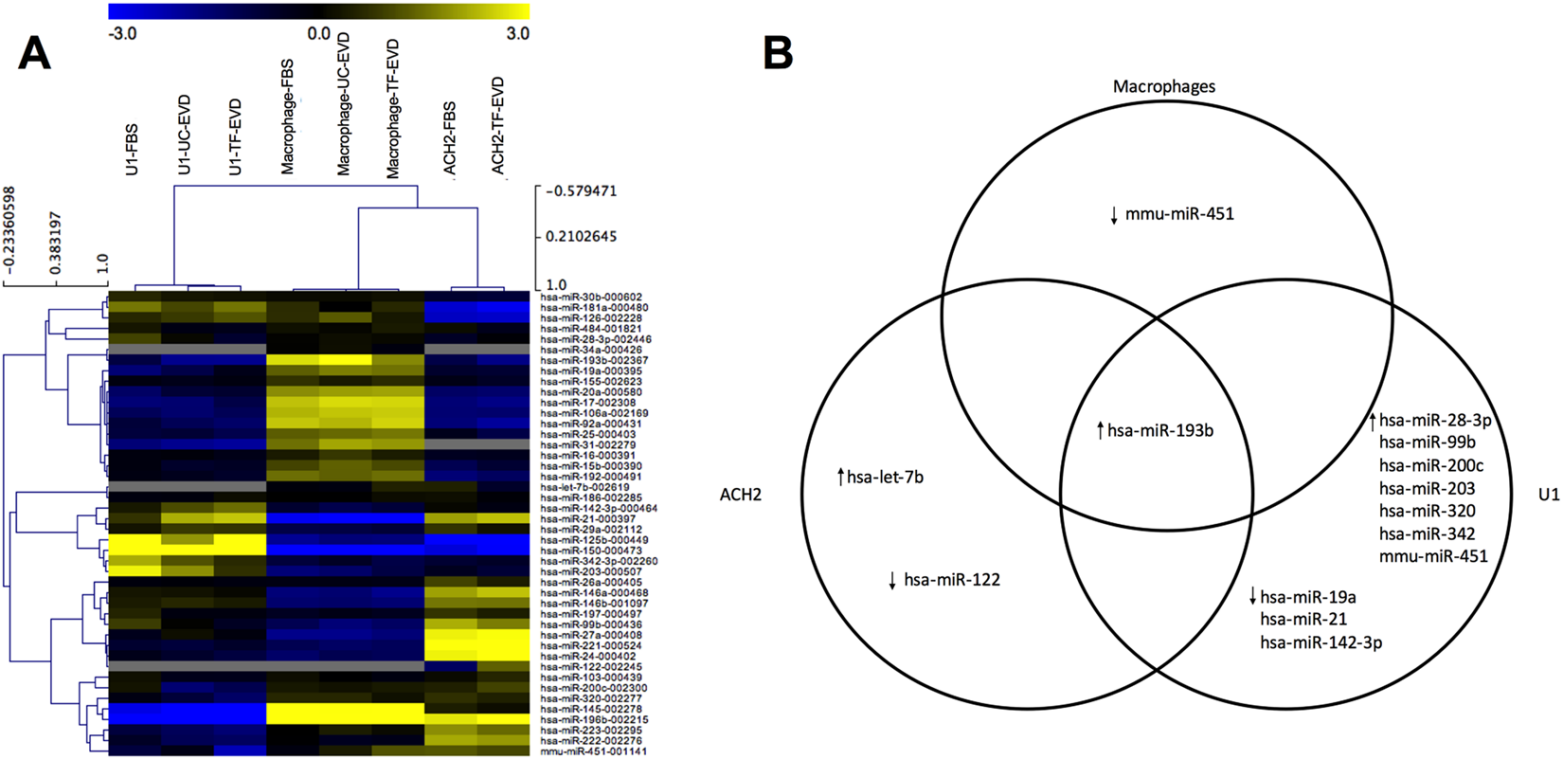
Little evidence for miRNA uptake from serum or serum-induced changes in miRNA expression. (**A**) Unbiased hierarchical clustering demonstrates cell type-dependent miRNA expression, but no indication of differences associated with TF-EVD medium conditions. (**B**) A total of five miRNAs appeared to be less abundant in TF-EVD conditions by two-fold or more in just one of the cell types, but with no consistent findings between cell types. miR-193b appeared to be 1.5 to 2-fold upregulated in all three cell types under depleted conditions.

Certain cellular microRNAs (miRNAs) have been reported to control retroviral replication^39, 40^, while exogenous RNAs in cell culture medium are said to be taken up by cells^41^. We hypothesized that miRNA levels in cultured cells might be augmented by miRNAs in serum particles including EVs, and that purported EV depletion processes might also indirectly affect miRNA expression. Either circumstance could result in reduced abundance of anti-HIV miRNAs, explaining an increase in HIV-1 production. However, results from miRNA profiling by a custom TaqMan low density array (TLDA) revealed no consistent changes in miRNAs across three cell types maintained in the different types of media. Unbiased hierarchical clustering showed cell type-specific miRNA expression patterns, but no indication of consistent differences associated with depleted serum conditions (Fig. 7A). A total of five miRNAs appeared to be less abundant by 2-fold or more in one cell type under depleted conditions (one in MDM, three in U1, one in ACH2), but no miRNA was 2-fold downregulated in more than one cell type, as one would expect if serum particles including EVs were an important and consistent source of miRNAs to supplement intracellular production (Fig. 7B). Importantly, previously reported anti-HIV miRNAs, including miRs-28-3p, -29a, -125b, -150, and -223, were not consistently diminished under depleted conditions. Similarly, there were no consistently upregulated miRNAs.

#### Gene expression

Gene array analysis was performed with cells grown in replete and depleted serum conditions. Gene ontology analysis of genes that were at least 2.5-fold more abundant under depleted media conditions revealed a significant increase in transcripts associated with lipid synthesis pathways, and especially sterol biosynthesis pathways (Table 1 and Table S1).

**Table 1:** Gene Ontology Analysis reveals upregulation of transcripts associated with lipid synthesis in cells grown in depleted serum conditions.

Term	Count	%	Benjamini-Hochberg
Steroid biosynthesis	8	1.4	4.1E-09
Sterol biosynthesis	6	1.0	8.3E-07
Endoplasmic reticulum	15	2.5	8.2E-07
Lipid synthesis	8	1.4	6.6E-07
Cholesterol biosynthesis	5	0.8	8.9E-06
Lipid metabolism	6	1.0	2.4E-03
Nucleosome core	4	0.7	9.5E-03
Steroid metabolism	4	0.7	9.6E-03

## Discussion

We show here an impact of serum processing protocols—which were designed and have been widely implemented for EV depletion—on HIV-1 production by susceptible cells. Additionally, HIV-1 latency models undergo a degree of viral activation under depleted serum conditions. Effects of depletion were absent or less pronounced in primary cells than in cell lines. Altogether, our findings suggest that EVs and other serum particles can inhibit HIV-1 infectivity and infection, as well as HIV-1 production by cell lines and primary leukocytes, and point to alterations in cellular lipid production as a possible underlying mechanism.

Increased HIV-1 production could be achieved in part through higher rates of infection (i.e., involving viral life cycle steps from cell binding through integration). The finding that certain cell adhesion molecules, including HIV-1 receptors and co-receptors, are upregulated on cells in depleted serum conditions might be consistent with more efficient infection. It could also explain the greater aggregation and syncytium formation we found in infected H9 and PM1 cell cultures. However, the observation that latency models are also affected by depleted serum conditions suggests that infection is not the sole explanation for our findings. Additionally, ultracentrifuge pellet add-back experiments suggest that any cellular responses to a decline in serum particles would have been reversed rapidly, perhaps too rapidly to invoke receptor involvement. Alternatively, virion interactions with EVs or lipids that were added back could have inhibited infection (although this seems inconsistent with previous findings)^42^.

Gene array data suggest another mechanism whereby HIV-1 production could be increased: in the relative absence of serum lipid-containing particles, lipids involved in EV (and thus virion) biogenesis are upregulated. EVs contain a high proportion of cholesterol, sphingomyelin and ceramide; therefore, transcribing genes involved in their biosynthesis may be a compensation response to perceived loss of exogenous sources, which must be sensed in some fashion. According to this hypothesis, normal interactions with diverse, non-self EVs or other lipid particles such as those found in serum preps could be viewed as a “security blanket” or a constant source of nutrition. Under conditions of lipid particle depletion, the cell might release its own EVs/particles but be unable to make up for the presence of foreign entities. This is feasible, as culture media tend to contain much lower amounts of EVs than unprocessed serum. The observed surface upregulation of adhesion receptors is also consistent with this idea, bringing cells into closer contact with other membranes (cellular or EV). Notably, the sterol upregulation scenario would imply that the increase in HIV release is non-specific, or specific only to the extent that virions bud from sterol-rich membrane microdomains. To delve further into these possibilities, more information is needed on how cells sense the presence of EVs and related particles: through molecules on the particle or cell surface, or both. One might also anticipate experiments to investigate the contribution of EVs and other particles from different cellular sources, which could explain the differing findings in the literature that we reviewed previously.

The latency model results are perhaps the most exciting, suggesting that serum particle depletion could inform new strategies in HIV-1 eradication therapy. Depleting bulk EVs/particles from plasma *in vivo* to increase HIV-1 production from latent cells in an eradication effort—in an approach analogous to leukapheresis—would be a difficult task. Even if such depletion were feasible, the same would likely be impossible in tissue. However, cell-particle signaling interactions could, hypothetically, be blocked through pharmacologic means. Clearly, more knowledge about the system is needed to assess this possibility and explore its implications.

We would like emphasize again that, although we use processes that are meant to deplete EVs from serum, other serum particles including lipoproteins and protein complexes may also be depleted, and to differing degrees. Thus, although EVs are likely involved in the effects we report, and we have attempted to follow recommendations and best practices in the EV field^43, 44^, EVs may not be the only or the most important contributors. There is growing recognition of co-depletion of non-EV lipids and proteins during EV depletion. Indeed, a recent publication from the Buzás lab found low-density lipoprotein contamination of EV preparations even after strict separation processes; furthermore, LDL particles decorated EVs after co-incubation^45^. It is possible that LDL, other lipoprotein particles, or protein aggregates contribute to the phenomena we report here. We hope that others will join us in pursuing the potentially informative studies that these findings might prompt, including:

1. Quantitation of the effects of intended EV depletion processes on other components of serum;
2. Examination of effects of co-depleted particles like LDL and HDL on HIV-1;
3. Functional assays including physically or chemically disrupted serum EVs, with care to confirm disruption of EVs as well as a lack of disruption of other potentially contributing lipid particles;
4. Breakdown of the serum EV complement by cell of origin, followed by functional experiments with highly purified EVs from cell culture.

## Electronic supplementary material

Supplementary Info
