# Supplementary material for: Serum extracellular vesicle depletion processes affect release and infectivity of HIV-1 in culture
Source: Sci Rep. 2017 May 31;7:2558. doi: 10.1038/s41598-017-02908-5 (PMC5451420; doi:10.1038/s41598-017-02908-5)
Supplement: Supplementary file 1 — Supplementary Info [file 41598_2017_2908_MOESM1_ESM.pdf]

Supplementary Materials

Serum extracellular vesicle depletion processes affect release and infectivity of HIV-1 in culture

Zhaohao Liao[1,#], Dillon C. Muth[1,3,#], Erez Eitan[4], Meghan Travers[3], Lisa N. Learman[3], Elin Lehrmann[4], Kenneth W. Witwer[1,2,3,*]

[1]Department of Molecular and Comparative Pathobiology, [2]Department of Neurology, and [3]Cellular and Molecular Medicine Program, The Johns Hopkins University School of Medicine, Baltimore, MD; [4]Laboratory of Neurosciences, National Institute on Aging, National Institutes of Health, 251 Bayview Boulevard, Baltimore, MD 21224.

[#] These authors contributed equally to this work

*Address correspondence to:

Kenneth W. Witwer, PhD

733 N. Broadway

Miller Research Building 829

Baltimore, MD 21205

Phone: 1-410-955-9770

Fax: 1-410-955-9823

Email: kwitwer1@jhmi.edu

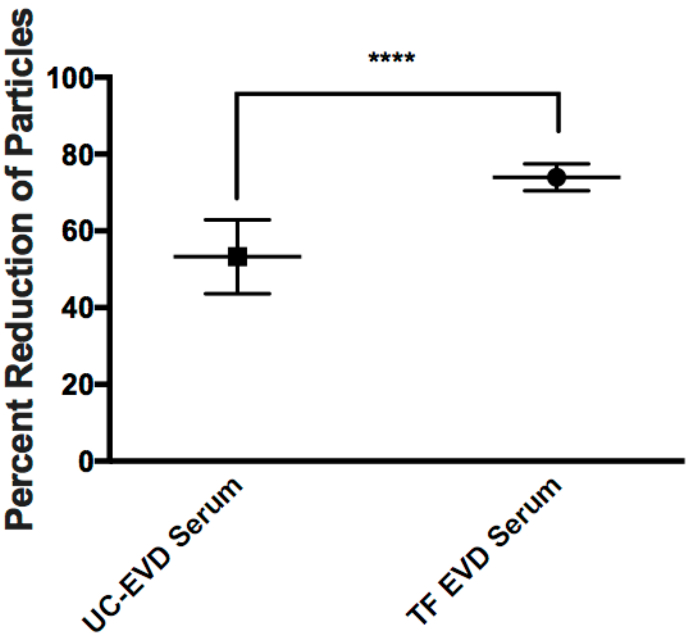

Figure S1. Particle depletion by ultracentrifugation and a proprietary commercial process. FBS lots depleted by ultracentrifugation as described in the Methods (UC-EVD, n=23) or by Thermo Fisher's proprietary process (TF-EVD, n=5) were compared with unmanipulated FBS by nanoparticle tracking. UC achieved approximately 40-60% reduction of total particle concentration, while the TF-EVD serum had an approximately 70-80% reduction. ****=p<.0001, unpaired t-test.

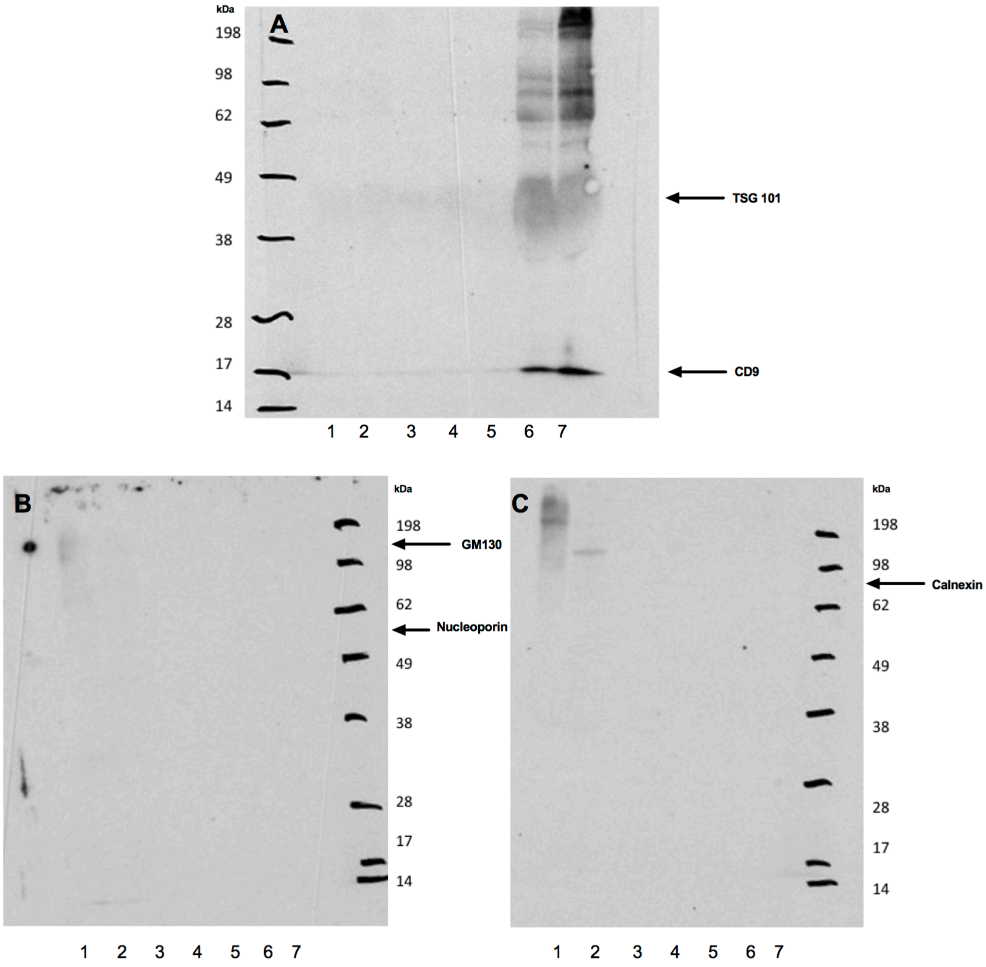

Figure S2. EV and cellular contaminant markers. A) Western Blot for TSG101 (~49kDa) and CD9 (~24kDa) shows dramatic enrichment of EV markers in UC pellets for both TF-source FBS (prior to depletion) and another FBS lot (lanes 6, 7) compared to the "source" and depleted FBS (Lanes 1-5 as in Figure 1). UC pellets (lanes 1, 2) examined for GM130 (~130kDa), Nucleoporin (~53kDa) and calnexin (~80kDa) (B, C) suggested little cellular contamination. These proteins were also not observed in FBS (lanes 5,6), UC-EVD media (lanes 3,4, same lots as above), or in TF-EVD (lane 7) media. All lanes were loaded with equal protein by mass.

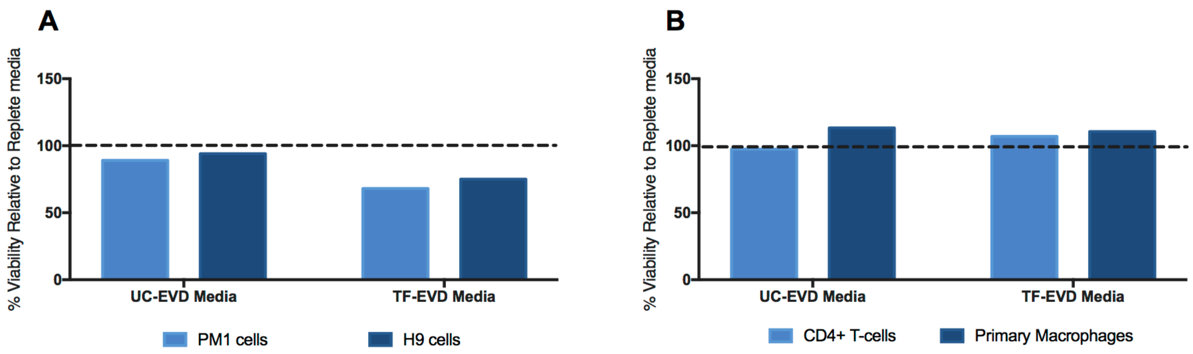

Figure S3. Cell Viability. A) LDH assay of uninfected H9 and PM1 cells grown in replete, UC-EVD and TF-EVD conditions after three days of incubation shows small decreases in cell viability, especially for cells grown in TF-EVD conditions. B) No significant differences for primary CD4+ T-cells or MDM. All: biological duplicates read in technical duplicate.

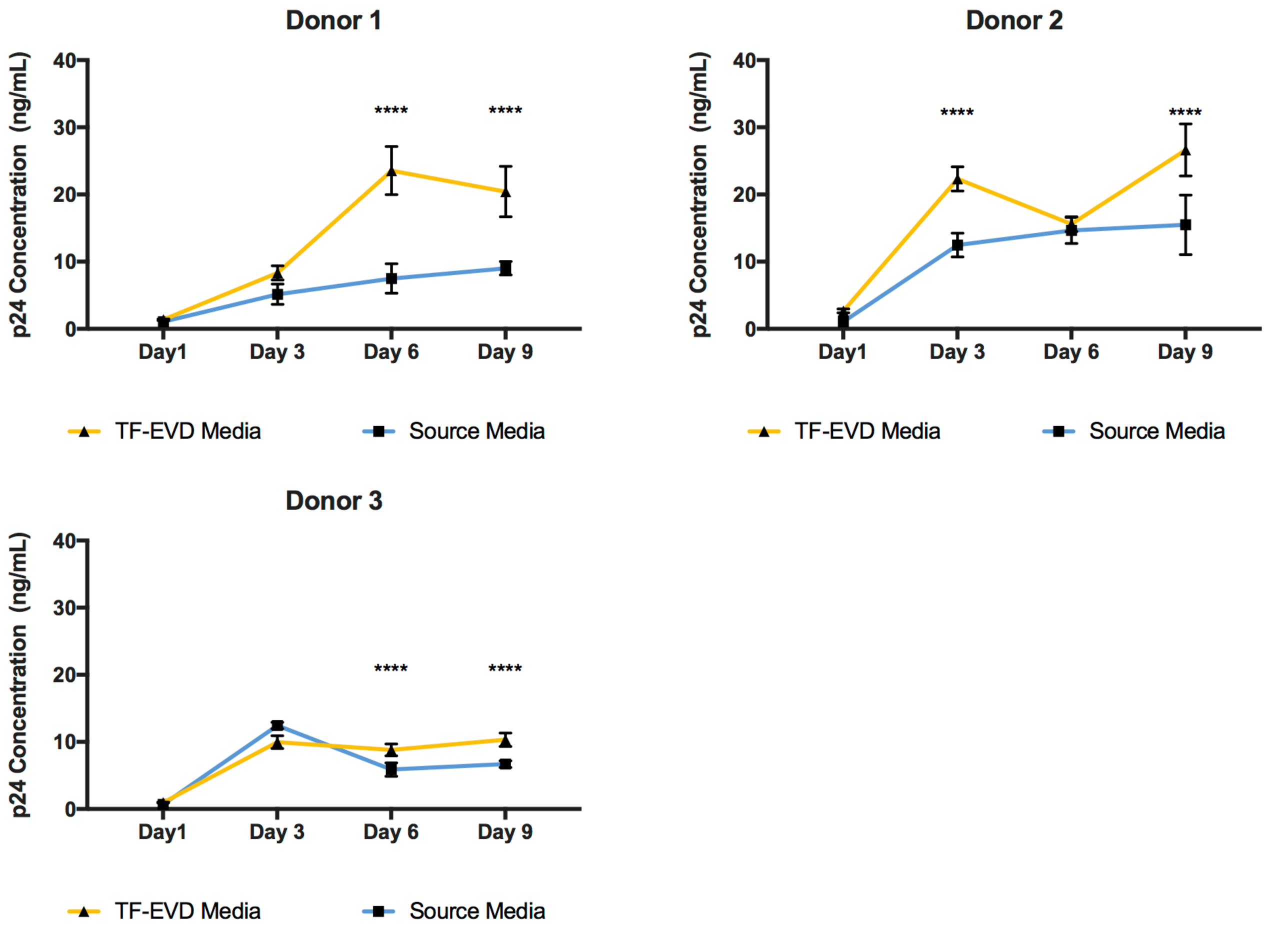

Figure S4. Individual experiments from Figure 3D, showing donor-to-donor variability of p24 production. All experiments included at least five culture replicates. For all graphs, ****=p>.0001, two-way ANOVA with Sidak's multiple comparison test.

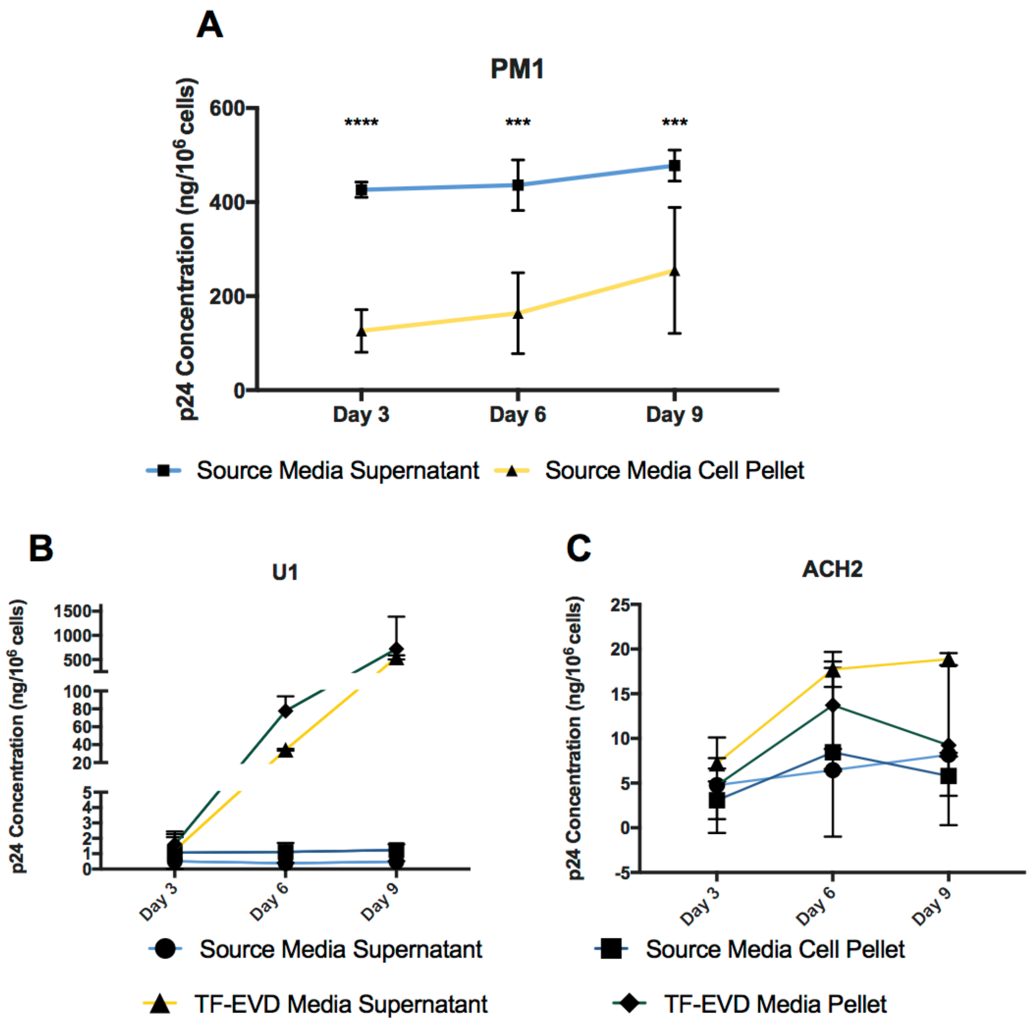

Figure S5. Cell-associated vs. released p24. A) In infected PM1 cell culture, significantly more p24 was released into the culture supernatant than was retained in the cells at days 3, 6, and 9 pi (with medium replacement at each time point; ****=p<0.001, **=p<.01, 2-way ANOVA with Sidak's multiple comparison test, n=3). B and C) At days 6 and 9 pi, there were similar amounts of p24 in culture supernatant and cells for both U1 (B) and ACH2 (C) latency models, with much lower levels in both compartments in cells grown in replete serum conditions.

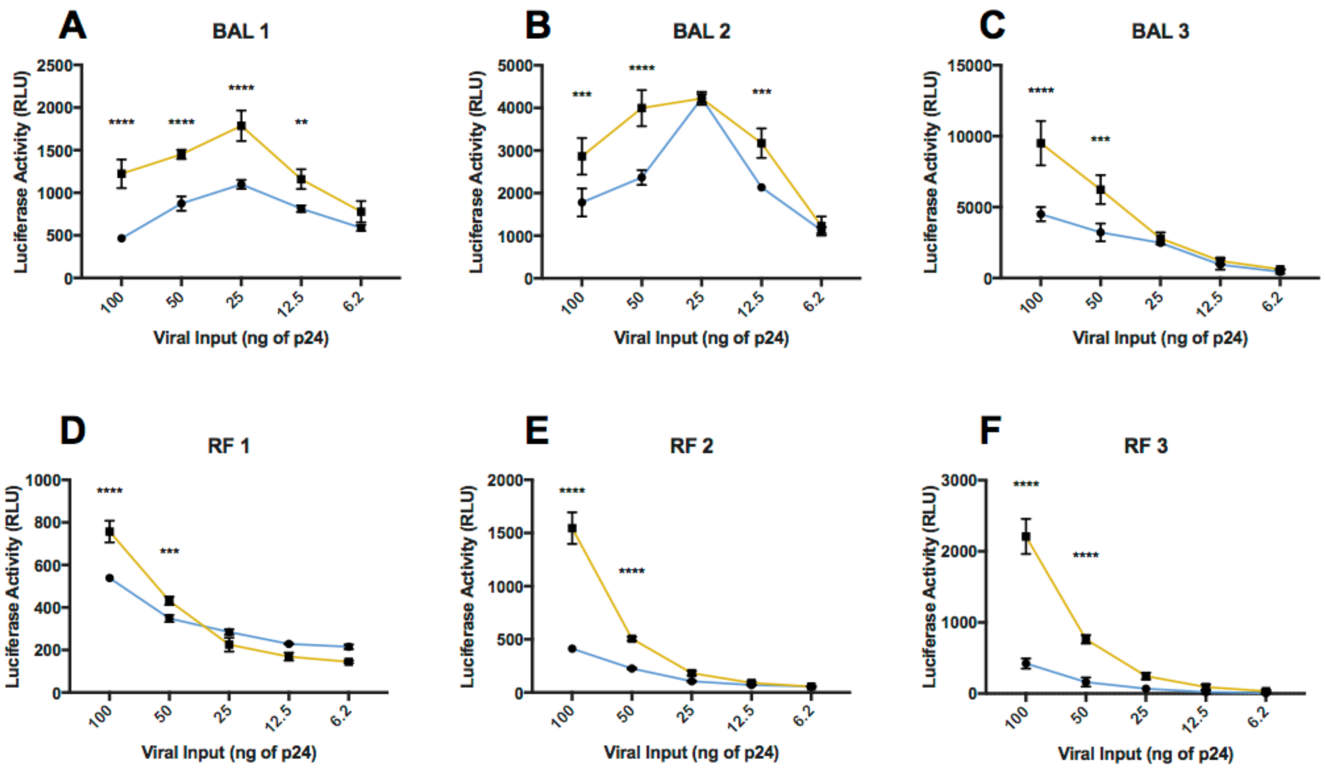

Figure S6. Infectivity: individual experiments represented in Figure 5A and B. Increased infectivity of virus produced under depleted serum conditions from PM1 (A-C) and H9 cells (D-F), as assessed by TZM-bl luciferase activity. Each independent experiment included three replicate culture wells; see Figure 5 for averages.

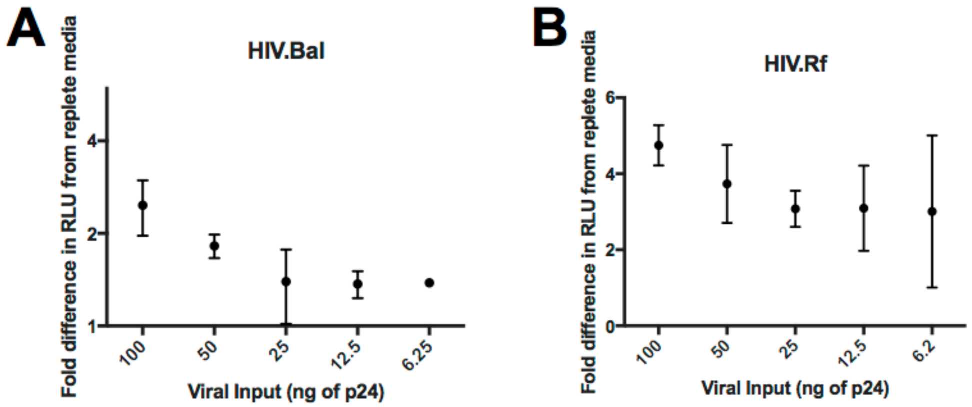

Figure S7. Representation of increased infectivity as fold change. Fold comparison of virus produced under EVD vs EVR conditions from PM1 (A) and H9 cells (B), as assessed by TZM-bl reporter cells. Shown are averages of the three independent experiments from Figure S6.

SREBF1
LDLR
CYP1A1
DHCR7
INSIG1
HMGCS1
HSD17B6
IDI1
FDFT1
HERPUD1
SGK1
SCD
LSS
LPIN1
ABCG1
RTN3
FOS
PTRF
SQLE
SDCBP
ACSL4
HIST2H2AA3
HIST1H2AC
HIST1H2BD
HIST1H2BJ
HIST1H3H

Table S1. Individual genes represented in Table 1.
